# Association of Pregestational BMI and Gestational Weight Gain with Maternal and Neonatal Outcomes in Adolescents and Adults from Mexico City

**DOI:** 10.3390/ijerph19010280

**Published:** 2021-12-28

**Authors:** Reyna Sámano, Gabriela Chico-Barba, María Eugenia Flores-Quijano, Estela Godínez-Martínez, Hugo Martínez-Rojano, Luis Ortiz-Hernandez, Oralia Nájera-Medina, María Hernández-Trejo, Cristopher Hurtado-Solache

**Affiliations:** 1 Coordinación de Nutrición y Bioprogramación, Instituto Nacional de Perinatología, Secretaría de Salud, Mexico City 11000, Mexico; maru_fq@yahoo.com (M.E.F.-Q.); eygodinez@hotmail.com (E.G.-M.); 2 Programa de Posgrado Doctorado en Ciencias Biológicas y de la Salud, División de Ciencias Biológicas y de la Salud, Universidad Autónoma Metropolitana, Mexico City 04960, Mexico; lortiz@correo.xoc.uam.mx (L.O.-H.); onajera@correo.xoc.uam.mx (O.N.-M.); 3 Escuela de Enfermería, Facultad de Ciencias de la Salud, Universidad Panamericana, Mexico City 03920, Mexico; 4 Programa de Maestría y Doctorado en Ciencias Médicas, Odontológicas y de la Salud, Universidad Nacional Autónoma de México, Mexico City 04510, Mexico; 5 Sección de Posgrado e Investigación de la Escuela Superior de Medicina del Instituto Politécnico Nacional, Mexico City 11340, Mexico; hmartinez_59@yahoo.com.mx; 6 Departamento de Atención a la Salud, Universidad Autónoma Metropolitana Xochimilco, Mexico City 04960, Mexico; 7 Departamento de Neurobiología del Desarrollo, Instituto Nacional de Perinatología, Secretaría de Salud, Mexico City 11000, Mexico; maria.h.trejo72@gmail.com; 8 Escuela de Ciencias de la Salud, Universidad del Valle de México-Chapultepec, Mexico City 11810, Mexico; cristopheralexis2324@hotmail.com

**Keywords:** adolescent pregnancy, perinatal outcomes, preeclampsia, anemia, Mexico

## Abstract

During pregnancy, adolescents experience physiological changes different from adults because they have not concluded their physical growth. Therefore, maternal and neonatal outcomes may not be the same. This paper aimed to analyze the association between pregestational BMI (pBMI) and gestational weight gain (GWG) with maternal and neonatal outcomes in adolescent and adult pregnant women. The authors performed an observational study that included 1112 women, where 52.6% (*n* = 585) were adolescents. Sociodemographic information, pBMI, GWG, neonatal anthropometric measures, and maternal and neonatal outcomes were obtained. Adolescent women had a mean lower (21.4 vs. 26.2, *p* ≤ 0.001) pBMI than adults and a higher gestational weight gain (12.3 vs. 10.7 kg, *p* ≤ 0.001). According to Poisson regression models, gestational diabetes is positively associated with insufficient GWG and with pregestational obesity. Furthermore, the probability of developing pregnancy-induced hypertension increased with pBMI of obesity compared to normal weight. Preeclampsia, anemia, and preterm birth were not associated with GWG. Insufficient GWG was a risk factor, and being overweight was a protective factor for low birth weight and small for gestational age. We conclude that pBMI, GWG, and age group were associated only with gestational diabetes and low birth weight.

## 1. Introduction

The Institute of Medicine (IOM) gestational weight gain guidelines recommend weight gain ranges for each pre-pregnancy body mass index (pBMI) category associated with a low prevalence of some maternal and neonatal adverse outcomes. These guidelines propose that adolescent pregnant women be categorized using BMI cutoff points for adults and be advised to gain within the same weight gain ranges [1]. Research has shown that some adolescents may stop or continue their physical growth during pregnancy [2], depending on their chronological age and energy stores [3,4]; this would also affect their perinatal outcomes [5]. The IOM recommendations take adolescent growth into account implicitly because lighter adolescents (possibly the younger ones) will most likely be categorized in the lower BMI group, and they would be recommended to gain weight at the highest range [1,6].

Regarding the evidence of the effects of pBMI and GWG on maternal and neonatal outcomes, it has been reported that being underweight prior to pregnancy increases the risk for preterm birth and for delivering a small for gestational age (SGA) newborn. On the other hand, overweight and obesity are high-risk factors for gestational diabetes, hypertensive syndrome, and fetal growth disorders. Concerning weight gain, women with insufficient gestational weight gain may experience anemia. Conversely, those with excessive weight gain are at an elevated risk of cesarean delivery, preeclampsia, gestational diabetes, blood transfusions, weight retention after delivery, and long-term obesity [7].

In addition, excessive GWG has been associated with childhood and adolescent overweight and obesity [8].

Mexico is within the top three Latin American countries with the highest rate of adolescent pregnancy [9]; among the countries of the Organization for Economic Co-operation and Development (OECD), Mexico is first in the rank [10]. According to national data, almost one out of five pregnancies in Mexico is adolescent [11].

In studies with Mexican adolescent women, pregestational BMI has been different from adults [5,12,13]. Moreover, being a pregnant adolescent increases the risk of complications, including preeclampsia, preterm birth, and low infant birth weight [14].

Unfortunately, insufficient and excessive gestational weight gains occur in two-thirds of pregnant women in different world regions [6,15,16]. For example, excessive weight gain has been reported in 33–41% of Mexican women, and inadequate gain in nearly 30% [10,17].

Like adults, adolescents are likely to experience excessive gestational weight gain, and they would also have a risk of having neonatal and maternal adverse outcomes [18]. Nevertheless, knowledge about perinatal maternal and neonatal outcomes in this age group is inconsistent [5,10,13,19]. Thus, it is expected that neonatal outcomes would also differ. Therefore, this study aimed to analyze the association between pregestational BMI and gestational weight gain with maternal and neonatal outcomes in adolescent and adult pregnant women.

## 2. Materials and Methods

We performed a cross-sectional study at the Instituto Nacional de Perinatología (INPer) in Mexico City. The INPer is a National Institute of Health that provides antenatal care to uninsured, low-income women with high-risk pregnancies. Pregnant adolescents (≤19 years old) and adult women (20–45 years old) were invited to participate in the study from 2013 to 2018. Sampling was non-probabilistic, and all consecutive women who met the following inclusion criteria were invited to participate: healthy singleton pregnancy, antenatal care and delivering her child in the INPer, and signed written informed consent. We excluded participants who consumed alcohol, tobacco, or drugs during pregnancy and those whose pregnancy resulted from rape. Participants were recruited during their outpatient consultation at the INPer.

### 2.1. Sociodemographic and Clinical Data

Age, educational level, occupation, and socioeconomic status were obtained through a questionnaire at the initial assessment. We obtained gestational age by ultrasound to define the trimester the participant initiated antenatal care at the INPer. Parity, history of disease, and type of delivery were obtained from clinical records.

### 2.2. Anthropometric Evaluation

Bodyweight was assessed at the beginning and the end of pregnancy. Pregestational weight was self-reported, and maximum gestational weight was obtained with a digital scale (TANITA, Tokyo, Japan, model BWB-800, accuracy 0.10 kg) one week before delivering. In addition, trained personnel obtained height measurement at first visit using the Lohman technique [20] with a stadiometer (SECA, Hamburg, Germany, model 208, accuracy 0.1 cm). pBMI was calculated using pregestational weight and the height from the first study visit. For adolescents, pBMI classification was obtained with AnthroPlus^®^ (World Health Organization, Geneva, Switzerland) according to percentiles: underweight < 3rd, normal weight 3–85th, overweight 85–97th, and obesity ≥ 97th [21]. Regarding adult women’s pBMI classification, pBMI was categorized as follows: underweight < 18.5, normal weight 18.5–24.99, overweight 25–29.99, obese ≥ 30 [1].

### 2.3. Gestational Weight Gain

We got the total gestational weight gain through the maximum gestational weight minus pregestational weight in kg. Then, according to the recommendations of the Institute of Medicine [22] of the United States of America, we classified the GWG based on each category of pBMI: underweight a gain of 12.5 ± 18 kg; normal weight a gain of 11.5 ± 16 kg; overweight a gain of 7 ± 11.5 kg; and obese a gain of 5 ± 9 kg. Then, GWG was divided into three categories: insufficient if the weight gain was below the recommendation; adequate if weight gain was within the recommendation; and excessive, when weight gain was above the recommendation.

### 2.4. Maternal Outcomes

Obstetricians registered pregnancy complications during prenatal visits to the INPer, and we obtained the information from the clinical records. Complications were identified and recorded in the following categories: gestational diabetes, pregnancy-induced hypertension, eclampsia/preeclampsia, and anemia.

According to medical procedures at the INPer, gestational diabetes was diagnosed between 24 and 28 gestational weeks by a glucose tolerance curve when one or more values were higher than expected—fasting glucose: ≥92 mg/dL, and 180 mg/dL at one hour or ≥153 at two hours, or ≥140 mg/dL at three hours, after a 75 g glucose charge. If the participants were diagnosed with diabetes before the week of gestation 24, they were considered as diabetes type 2 [23] and were excluded from the analysis. Pregnancy-induced hypertension was defined as systolic blood pressure > 140 mm/Hg and diastolic blood pressure > 90 mm/Hg [24,25]. Preeclampsia was defined as the presence of new-onset hypertension and proteinuria occurring after 20 weeks gestation, whereas eclampsia was defined as the development of seizures in a woman with preeclampsia [25]. Anemia was classified when women had <12.3 g/dL during the second or/and third trimester; we used an altitude correction for hemoglobin of +1.3 g/dL [26].

### 2.5. Neonatal Outcomes

Gestational age was obtained by the Capurro method and recorded in weeks and days. If the gestational age was under 36.6 weeks, it was classified as preterm; the gestational age between ≥37 and ≤42 weeks was identified as a term delivery.

We measured and recorded weight (SECA 374, model “Baby and Mommy”; accuracy 0.1 g) and length (stadiometer SECA 416; accuracy 0.1 cm) at birth. Then, low birth weight (LBW) was defined as <2500 g and small for gestational age (SGA) according to weight for gestational age of Mexican children [27].

### 2.6. Ethical Aspects

This research was approved by the Institutional Ethics, Biosafety, and Research Committees (registration numbers 212250-494811 and 2017-2-101). All adults, adolescents, and guardians were informed of the study’s objectives and the procedures involved. Confidentiality was guaranteed by assigning a folio number during the participant’s data collection and its analysis.

### 2.7. Statistical Analysis

We performed univariate analysis to describe the sample characteristics. Categorical variables were presented as frequencies and percentages, and continuous variables as mean ± standard deviation or median (p25–p75). We compared the frequencies of sociodemographic, maternal, and neonatal characteristics between adolescents and adults using the Chi-square test in the bivariate analysis. Unadjusted Poisson regression models were performed to analyze the association of GWG, age, pBMI as independent variables with maternal and neonatal outcomes as dependent variables. We ran three different models: in M1, the variables GWG and pBMI were introduced in different (independent) models; in M2, both variables, GWG and pBMI, were included in the same model as main effects; and in M3, both variables, GWG and pBMI, were included in the same model, with interaction with age.

All models were adjusted by socioeconomic level, initiation of prenatal care, parity, and history of diseases. The reference groups were adolescents, adequate GWG, and normal weight. Statistical significance was considered with a *p*-value < 0.050. All analyses were performed using the software Stata/v.SE16.1 (College Station, TX, USA) [28].

## 3. Results

### 3.1. Participants’ Characteristics

A total of 1112 pregnant women participated in the study, where 52.6% (*n* = 585) of them were adolescents. The mean age for adolescents was 15.9 ± 1.4 years and 30.2 ± 6.2 years for adult participants. Table 1 shows that all sociodemographic and maternal characteristics were statistically different between age groups. Lower educational levels and being a student were more frequent among adolescents than in adults. Further, most adolescents initiated prenatal care in the second trimester, compared to adults who sought prenatal care in the first trimester. Pre-pregnancy BMI was lower in adolescents than adult mothers (21.4 vs. 26.2, *p* ≤ 0.001). In contrast, the adolescent group had higher gestational weight gain than other participants (12.3 vs. 10.7 kg, *p* ≤ 0.001). Pregestational overweight and obesity and excessive gestational weight gain were more common in pregnant adults than in adolescents. Gestational diabetes was more frequent within adult women than in adolescents. Overall, median hemoglobin was 14.00 g/dL; for adolescents it was 13.89 g/dL and 14.1 g/dL for adults (*p* = 0.003). However, the medians for both groups indicated a normal level of hemoglobin. Regarding the newborns, the mean birth weight was 2918 ± 510 g, and the median gestational age was 39 (min 26–max 40) weeks; (data not shown in table nor figures). Meanwhile, the percentage of preterm birth was higher in adult mothers (*p* = 0.035) Table 1.

**Figure 1:**
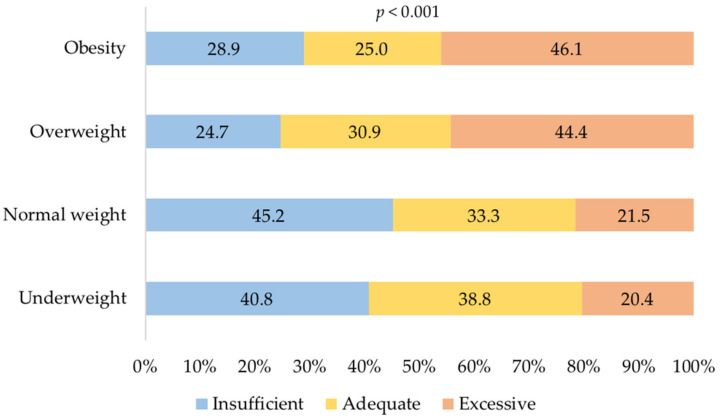
Gestational weight gain by pregestational body mass index.

The GWG in categories was associated with pregestational BMI (*p* < 0.001), as seen in Figure 1. Excessive GWG was more frequent among women with overweight and obesity than in women with normal weight. Similar figures were observed when the association was stratified by the age group (data not shown).

### 3.2. Maternal Outcomes

Table 2 shows the Poisson regression models for gestational diabetes as the outcome variables. When GWG and pBMI were independently analyzed, we did not find any statistically significant association of the studied variables with gestational diabetes (M1). In Model 2, with main effects analysis, the probability of gestational diabetes was marginally observed (*p* = 0.053) for those with insufficient GWG. Factors associated with a likelihood of having gestational diabetes were having obesity (PR: 1.54, *p* = 0.038) and being an adult (PR: 1.81, *p* = 0.004) (M2). The interaction of GWG and age was statistically significant; the adults with insufficient weight gain had a 2.59 probability of having gestational diabetes than those with an adequate GWG.

Nevertheless, excessive GWG showed no association with gestational diabetes in adults. Likewise, no association with GWG was found in adolescents. Moreover, those participants with pregestational obesity had a 1.59 probability of gestational diabetes (M3).

For the other maternal outcomes that we assessed, only the participants with obesity had a greater probability of pregnancy-induced hypertension than normal weight. We found no associations with preeclampsia nor anemia. Further, we did not observe any interaction between variables, as can be seen in Table 3.

### 3.3. Neonatal Outcomes

Regarding neonatal outcomes, Table 4 shows that neither age group, GWG, nor pBMI were associated with preterm birth; only being an adult showed a marginal association compared to adolescents (RP: 1.44, *p* = 0.079).

The factor that increased the probability of low birth weight was insufficient GWG (RP: 1.61, *p* = 0.008). Contrarily, pregestational overweight marginally decreased the probability of having a low birth weight baby (M2).

Similar results were observed for the small for gestational age outcome. In M2, insufficient GWG marginally increased the probability of SGA (RP: 1.36, *p* = 0.084), but pregestational overweight decreased the probability (RP: 0.49, *p* = 0.007).

## 4. Discussion

This investigation identified that GWG and age are risk factors for maternal and neonatal outcomes. Our study highlights that insufficient GWG and pre-pregnancy BMI were associated with gestational diabetes mellitus. Contrary to expectations, the adult women group was associated with gestational diabetes but not with preeclampsia.

In our study, more adolescent women started pregnancy with an adequate BMI while more adult women had pregestational overweight or obesity. By the end of gestation, the younger group had a higher GWG in kg compared to adults, as had been previously observed in other hospital-based studies with Mexican and Chinese women [5,29]. These observations are consistent with the IOM presumption that adolescents, especially the younger ones, would more likely be categorized in a “lighter group” and thus be advised to gain more weight [1,29].

Three out of ten participants had adequate GWG, which was more frequent among adolescent women than adults; this information was similar to a US study [30]. In contrast, other publications did not observe differences in the distribution of GWG categories between age groups [31]. Variations in the percentages of GWG categories in the different studies may be due to the availability of antenatal care and ethnic and racial differences, ranging from 50% to 61% for excessive GWG and from 20% to 30% for adequate [29].

Our results showed that the cesarean-section rate was higher than the recommended by the WHO [32]. The Latin American region has the highest rates of cesarean section worldwide [5,33]. C-section increases healthcare costs and negatively affects exclusive breastfeeding [34].

### 4.1. Maternal Outcomes

Unlike other studies [19,35,36], our research showed that the frequency of anemia was similar among adolescents and adults. Nevertheless, our results agree with a study performed with Mexican women [5]. Regarding anemia, we only have a marginal statistically significant association with pregestational low weight. However, in other studies, pregestational low weight has been associated with an increased risk of anemia [37,38]. Therefore, it could be pertinent to highlight the concern and importance of timely antenatal care, especially in adolescent women.

#### 4.1.1. Gestational Diabetes

Our study showed a higher frequency of gestational diabetes in adults and even in adolescent women, compared to data from Mexican studies [5,39], where the prevalence ranged from 1% to 3.4% compared with the 14.5% from our research. The discrepancy may be explained since our study took place at a tertiary care center, where complicated pregnancies are referred.

We found that among the adult participants, pregestational obesity was associated with a higher risk of gestational diabetes. However, in adolescent women, this relationship was not observed. Our findings highlighted that gestational diabetes was higher among adult women who started pregnancy with pregestational obesity and adults with insufficient GWG. These findings coincide with other research showing that insufficient GWG among women with prior diabetes and gestational diabetes is up to 50%, and excessive GWG is 20% in Hispanics [40] and Anglo-Saxon women [41]. An explanation for the insufficient GWG is that women diagnosed with gestational diabetes in weeks 24–28 are likely to change their habits, and less gestational weight gain is recommended after diagnosis [42]. In this sense, a healthy lifestyle is encouraged for pregnant women, and GWG is timely monitored, resulting in insufficient weight gain [43]. According to the IOM, in first-level care hospitals, personalized attention is offered focused on the care women should have to avoid complications, resulting in insufficient GWG in the overweight and obese women [44]. In the INPer, almost all women are diagnosed in the 24–28 weeks of gestation and have personalized antenatal care, which coincides with previous statements. 

#### 4.1.2. Preeclampsia

Preeclampsia is a disease that is more common in adolescents than in adult women, and it has a high maternal–fetal mortality rate [45]. However, the rate of preeclampsia in our study (5%) was similar in both age groups (adolescents vs. adults), but it was higher than the reported in a multicentric study [35], in specialized care [46] or tertiary care centers [45]. None of the risk factors that we assessed in our study were associated with preeclampsia, possibly because of other risk factors [47] that we did not take into account in our study, such as the maternal family history of preeclampsia, polymorphisms, or nutrition-related factors previously associated with this complication [48].

Our results show that none of the models’ independent or adjusting variables, such as the initiation of timely antenatal care, were associated with pregnancy-induced hypertension. According to the evidence, the initiation of timely antenatal care prevents different pregnancy complications [45], which may be risk factors for preeclampsia [49].

Pregestational obesity increased the probability of pregnancy-induced hypertension, similar to findings from other studies [50,51]. This association may be explained by the link with the secretion of proinflammatory cytokines that are associated with adverse events [51].

#### 4.1.3. Anemia

Anemia frequency was similar in adolescent and adult women in our study. We found a marginal association of anemia with low weight pBMI (*p* = 0.086), but maternal age and GWG were not associated. In white women from the USA, pre-pregnancy obesity was not associated with anemia. Anemia was less frequent in women with obesity than normal weight; the authors conclude that the excess weight can sequester iron of the stores [52]. Regarding GWG in a study performed with Brazilians, although 30% was supplemented with iron and folic acid, their prevalence of anemia was 17%. Furthermore, they did not find any association between inadequate GWG (excessive or insufficient) and anemia [53]. In Mexico, supplementation of iron in either of the schemes daily or weekly has effectively prevented anemia among pregnant women and low birth weight in their neonates [54].

According to data obtained from the INPer patients, anemia in pregnant adolescents has decreased from 80% in 2003 [55] to 38% in 2012 [56]. Our results show even a lower frequency of 10%. In Mexico, according to the national health norm NOM-007 [57], all pregnant women should receive prophylactic and therapeutic iron supplementation from the second trimester of gestation. Although we did not evaluate compliance with supplementation recommendations, we may infer that this maneuver could have resulted in the low percentage of anemia found in our study. If this is correct, we could document that antenatal care in this institutional environment would effectively prevent some adverse perinatal outcomes due to the low frequency of anemia in our sample. On the other hand, a third of the pregnancies have low pregestational weight and anemia; this combination is associated with adverse perinatal outcomes [58].

### 4.2. Neonatal Outcomes

Our data showed that adolescent and adult mothers had 10.4% and 14.0% of preterm neonates, respectively. Other multi-country studies reported different results than ours [35,59], where the higher figures among those <15 years old were nearing 7%, which was lower than our results. In Japan, preterm prevalence was not different between adolescents and adults, although the prevalence reached 20% [60]. The discrepancies in the frequency of preterm birth may be explained by socio-cultural context and the quality of antenatal care from each region.

A multicentric study showed that younger adolescents had a higher prevalence of low birth weight [35]. Nevertheless, in our study adolescents and adults had similar frequencies of low birth weight neonates, although the Poisson regression models showed that adults with insufficient GWG have a higher probability of low birth weight. However, there was a marginal association. Similar data were found in other studies [58,59], highlighting other associated factors such as age and pBMI [61].

Adolescents in our study had a higher frequency of small for gestational age babies compared to adult mothers, these data coincide with other research [5,59]. The authors highlight their concern of inadequate antenatal care as a possible explanation [5]. Women under 19 years frequently initiate care later than adults [62], which delays and limits professional advice to prevent pregnancy complications. Another factor associated with small-for-gestational-age during adolescence is the competence for nutrients between adolescents and their fetuses [63].

Regarding pregestational overweight as a protective factor to small-for-gestational-age neonates, previous research has directly related pBMI and intrauterine growth [64]. However, women with pre-pregnancy excess weight are more prone to have macrosomic or large-for-gestational-age babies [65,66,67], which is more common among adult women. In addition, adults with high pBMI have a higher risk of gestational diabetes mellitus, resulting in more adverse neonatal outcomes [68]. The above argument could explain pregestational overweight as a protective factor for small-for-gestational-age babies in our study. On the other hand, we had a few large-for-gestational-age and macrosomia neonate cases. Maybe, for this reason, these variables were not significant in the different models.

### 4.3. Limitations and Strengths

Our research has some limitations. We did not assess sociodemographic, clinical, and mental health (depression, network support during pregnancy) characteristics that may play a role in the perinatal outcomes. Further, our sampling was consecutive and convenient; therefore, our results cannot be generalized to all Mexican pregnant women. Nevertheless, our results open the line to other research exploring other variables in a more representative sample that compares among more than two age groups (e.g., >35 age pregnant women). On the other hand, we did not have the cesarean-section delivery election reasons, whose frequency was high in our sample. Another limitation is that we used self-reported pre-pregnancy weight which could introduce bias; however, evidence supports the use of self-reported weight as a cost-effective and practical measurement [69].

One strength of our study was the similar number of adolescents and adult women and a wide range of ages, which allowed us to have a more complex context of the maternal and neonatal outcomes of a sample of women from a tertiary care center. Besides this, in the adolescent group, we classified pBMI according to parameters suitable for their age. Thus, our research updates this topic from a developing country with a high rate of teenage pregnancy, overweight, and obesity in the female population. Although there is much evidence regarding pBMI, GWG, and perinatal outcomes, another strength of our study is that we found that gestational diabetes is also high in pregnant adolescents, which we did not expect. In this sense, timely interventions are needed in this age group because pregnancy impacts the adolescents’ growth [4] and is probably determining their future metabolic health and their offspring’s. Moreover, teenage mothers usually get pregnant later on, and having had gestational diabetes makes them prone to more maternal and neonatal complications.

## 5. Conclusions

In a sample of pregnant Mexicans who received antenatal care at a tertiary care center, pregestational BMI was associated with gestational diabetes and pregnancy-induced hypertension. An insufficient GWG and being an adult woman were associated with the probability of gestational diabetes and, marginally, with anemia.

This study was carried out in a tertiary hospital, where pregnant women with a higher risk of complications and adverse perinatal outcomes, such as adolescents or starting pregnancy with low or high BMI, are treated. Therefore, it was important to describe how a modifiable factor, such as gestational weight gain, modifies the risk of such adverse outcomes.

The study results confirm the need to create environments and promote strategies that prevent adolescent pregnancy and promote lifestyles that help women of reproductive age start pregnancy with adequate and healthy weight and provide the necessary advice and support to promote adequate weight gain.

## Figures and Tables

**Table 1:** Demographic and clinical characteristics of pregnant women and their newborns.

		Adolescents	Adults	Total	*p*-Value
Variables		*n* (%)	*n* (%)	*n* (%)	
Sociodemographic	Education level				
	Elementary school	153 (26.1)	25 (4.7)	178 (16.0)	<0.001
	Junior high	358 (61.2)	139 (26.4)	497 (44.7)	
	High school	71 (12.1)	226 (42.9)	297 (26.7)	
	College	3 (0.5)	137 (26.0)	140 (12.6)	
	Occupation				
	Homemaker	465 (79.5)	438 (83.1)	903 (81.2)	<0.001
	Student	104 (17.8)	6 (1.1)	110 (9.9)	
	Employee	16 (2.7)	83 (15.8)	99 (8.9)	
	Socioeconomic level				
	Low	247 (42.2)	409 (77.6)	113 (100)	<0.001
	Middle	237 (40.5)	106 (20.1)	343 (100)	
	High	101 (17.3)	12 (2.3)	656 (100)	
Maternal	Initiation of prenatal care				
	1st trimester	193 (33.0)	284 (53.9)	477 (42.9)	<0.001
	2nd trimester	328 (56.1)	196 (37.2)	524 (47.1)	
	3rd trimester	64 (10.9)	47 (8.9)	111 (10.0)	
	Pre-pregnancy BMI				
	Underweight	29 (5.0)	20 (3.8)	49 (4.4)	<0.001
	Normal weight	430 (73.5)	230 (43.6)	660 (59.4)	
	Overweight	67 (11.5)	156 (29.6)	223 (20.1)	
	Obesity	59 (10.1)	121 (23.0)	180 (16.2)	
	Gestational weight gain				
	Insufficient	214 (36.6)	211 (40.0)	425 (38.2)	0.022
	Adequate	207 (35.4)	146 (27.7)	353 (31.7)	
	Excessive	164 (28.0)	170 (32.3)	334 (30.0)	
	Parity				
	Primigravida	521 (89.1)	271 (51.4)	792 (71.2)	<0.001
	Multigesta	64 (10.9)	256 (48.6)	320 (28.8)	
	Cesarean section	272 (46.5)	367 (69.9)	639 (57.6)	<0.001
	History of disease	137 (23.4)	192 (36.4)	329 (29.6)	<0.001
	Complications during pregnancy				
	Gestational diabetes	53 (9.1)	111 (21.1)	164 (14.7)	<0.001
	Preeclampsia	34 (5.8)	31 (5.9)	65 (5.8)	0.960
	Pregnancy-induced hypertension	54 (9.2)	48 (9.1)	102 (9.2)	0.944
	Anemia ^1^	44 (9.4)	59 (11.5)	103 (10.5)	0.274
Neonate	Preterm	61 (10.4)	77 (14.6)	138 (12.4)	0.035
	Low birth weight	94 (16.1)	87 (16.5)	181 (16.3)	0.843
	Small for gestational age	102 (17.4)	72 (13.7)	174 (15.6)	0.084
	Macrosomia	3 (0.5)	11 (2.1)	14 (1.3)	0.019
	Large for gestational age	15 (2.6)	27 (5.1)	42 (3.8)	0.025

^1^*n* = 983.

**Table 2:** Poisson regression models for the association of GWG and pBMI with gestational diabetes as the outcome variable.

	M1		M2		M3	
Variable	PR	*p*-Value	PR	*p*-Value	PR	*p*-Value
Age group						
Adults	-	-	1.81	0.004	1.06	0.862
GWG						
Insufficient	1.41	0.080	1.46	0.053	0.80	0.490
Excessive	1.22	0.344	1.13	0.574	0.83	0.592
Interaction of GWG with age						
Insufficient	-	-	-	-	2.59	0.023
Excessive	-	-	-	-	1.67	0.239
pBMI						
Underweight	0.70	0.489	0.71	0.503	0.71	0.514
Overweight	1.21	0.342	1.29	0.218	1.30	0.196
Obesity	1.45	0.065	1.54	0.038	1.59	0.028

GWG: Gestational Weight Gain, pBMI: pregestational body mass index, M1: model 1, M2: model 2, M3: model 3, PR: prevalence ratio. In M1, the variables GWG and pBMI were introduced in different (independent) models. In M2, both variables, GWG and pBMI, were included in the same model as main effects. In M3, both variables, GWG and pBMI, were included in the same model, with interaction with age. All models were adjusted by socioeconomic level, initiation of prenatal care, parity, and history of diseases. The reference groups were adolescents, adequate GWG, and normal weight.

**Table 3:** Poisson regression models for the association of GWG and pBMI with preeclampsia, pregnancy-induced hypertension, and anemia.

	M1		M2	
Variable	PR	*p*-Value	PR	*p*-Value
Preeclampsia				
Age group				
Adults	-	-	0.82	0.500
GWG				
Insufficient	0.68	0.225	0.68	0.217
Excessive	1.06	0.839	0.98	0.948
pBMI				
Underweight	0.00	0.981	0.00	0.981
Overweight	1.00	0.993	0.92	0.818
Obesity	1.68	0.095	1.58	0.153
Pregnancy-induced hypertension				
Age group				
Adults	-	-	0.67	0.092
GWG				
Insufficient	0.73	0.219	0.74	0.239
Excessive	1.20	0.449	1.09	0.718
pBMI				
Underweight	0.23	0.142	0.23	0.140
Overweight	1.17	0.564	1.07	0.796
Obesity	1.84	0.015	1.70	0.039
Anemia				
Age group				
Adults	-	-	1.10	0.698
GWG				
Insufficient	0.98	0.938	0.96	0.855
Excessive	0.70	0.178	0.76	0.296
pBMI				
Underweight	1.87	0.082	1.86	0.086
Overweight	0.78	0.359	0.82	0.465
Obesity	0.65	0.168	0.69	0.249

GWG: Gestational Weight Gain, pBMI: pregestational body mass index, M1: model 1, M2: model 2, PR: prevalence ratio. In M1, the variables GWG and pBMI were introduced in different (independent) models. In M2, both variables, GWG and pBMI, were included in the same model as the main effects. All models were adjusted by socioeconomic level, initiation of prenatal care, parity, and history of diseases. The reference groups were adolescents, adequate GWG, and normal weight.

**Table 4:** Poisson regression models for the association of GWG and pBMI with preterm birth, small for gestational age and low birth weight.

	M1		M2	
Variable	PR	*p*-Value	PR	*p*-Value
Preterm				
Age group				
Adults	-	-	1.44	0.079
GWG				
Insufficient	1.35	0.141	1.33	0.157
Excessive	0.82	0.401	0.83	0.430
pBMI				
Underweight	0.99	0.972	0.99	0.987
Overweight	0.80	0.343	0.90	0.661
Obesity	0.88	0.615	0.98	0.951
Low birth weight				
Age group				
Adults	-	-	1.11	0.579
GWG				
Insufficient	1.67	0.004	1.61	0.008
Excessive	0.91	0.679	0.97	0.906
pBMI				
Underweight	0.85	0.665	0.87	0.704
Overweight	0.58	0.015	0.66	0.066
Obesity	0.66	0.071	0.73	0.177
Small for gestational age				
Age group				
Adults	-	-	0.82	0.305
GWG				
Insufficient	1.43	0.046	1.36	0.084
Excessive	0.84	0.429	0.92	0.704
pBMI				
Underweight	1.18	0.609	1.19	0.579
Overweight	0.44	0.002	0.49	0.007
Obesity	0.72	0.155	0.77	0.284

GWG: Gestational Weight Gain, pBMI: pregestational body mass index, M1: model 1, M2: model 2, M3: model 3, PR: prevalence ratio. In M1, the variables GWG and pBMI were introduced in different (independent) models. In M2, both variables, GWG and pBMI, were included in the same model as main effects. All models were adjusted by socioeconomic level, initiation of antenatal care, parity, and history of diseases. The reference groups were adolescents, adequate GWG, and normal weight.

## Data Availability

The data presented in this study are available from the corresponding author upon reasonable request.

## Abbreviations

GWG	Gestational weight gain
BMI	Body mass index
pBMI	Pregestational body mass index
IOM	Institute of Medicine
INPer	National Institute of Perinatology
USA	United States of America
WHO	World Health Organization
